# An Improved Gaussian Mixture Model for Damage Propagation Monitoring of an Aircraft Wing Spar under Changing Structural Boundary Conditions

**DOI:** 10.3390/s16030291

**Published:** 2016-02-26

**Authors:** Lei Qiu, Shenfang Yuan, Hanfei Mei, Fang Fang

**Affiliations:** The State Key Lab of Mechanics and Control of Mechanical Structures, Nanjing University of Aeronautics and Astronautics, 29 Yu Dao Street, Nanjing 210016, China; lei.qiu@nuaa.edu.cn (L.Q.); meihanfei@nuaa.edu.cn (H.M.); fang@nuaa.edu.cn (F.F.)

**Keywords:** aircraft structure, structural health monitoring, guided wave, damage monitoring, Gaussian mixture model, time-varying condition

## Abstract

Structural Health Monitoring (SHM) technology is considered to be a key technology to reduce the maintenance cost and meanwhile ensure the operational safety of aircraft structures. It has gradually developed from theoretic and fundamental research to real-world engineering applications in recent decades. The problem of reliable damage monitoring under time-varying conditions is a main issue for the aerospace engineering applications of SHM technology. Among the existing SHM methods, Guided Wave (GW) and piezoelectric sensor-based SHM technique is a promising method due to its high damage sensitivity and long monitoring range. Nevertheless the reliability problem should be addressed. Several methods including environmental parameter compensation, baseline signal dependency reduction and data normalization, have been well studied but limitations remain. This paper proposes a damage propagation monitoring method based on an improved Gaussian Mixture Model (GMM). It can be used on-line without any structural mechanical model and *a priori* knowledge of damage and time-varying conditions. With this method, a baseline GMM is constructed first based on the GW features obtained under time-varying conditions when the structure under monitoring is in the healthy state. When a new GW feature is obtained during the on-line damage monitoring process, the GMM can be updated by an adaptive migration mechanism including dynamic learning and Gaussian components split-merge. The mixture probability distribution structure of the GMM and the number of Gaussian components can be optimized adaptively. Then an on-line GMM can be obtained. Finally, a best match based Kullback-Leibler (KL) divergence is studied to measure the migration degree between the baseline GMM and the on-line GMM to reveal the weak cumulative changes of the damage propagation mixed in the time-varying influence. A wing spar of an aircraft is used to validate the proposed method. The results indicate that the crack propagation under changing structural boundary conditions can be monitored reliably. The method is not limited by the properties of the structure, and thus it is feasible to be applied to composite structure.

## 1. Introduction

Structural Health Monitoring (SHM) system was proposed to be a key part of Prognosis and Health Management (PHM) system and Integrated Vehicle Health Management (IVHM) system to reduce the maintenance cost and meanwhile ensure the operational safety of aircraft structures [1,2,3,4]. It utilizes advanced sensors to monitor the health and damage state of structures so as to inform decisions regarding maintenance and logistics to reduce the need for human intervention so as to avoid unnecessary maintenance events and maintain the structural safety [3,4].

In recent decades, SHM technology has gradually developed from theoretic and fundamental research to aerospace engineering applications [5,6], in which, the problem of reliable damage monitoring under time-varying conditions has become one of the main obstacles for applying SHM technology to real aircraft structures [7,8,9]. Real aircraft structures usually serve under uncertain and non-linear time-varying conditions such as environmental conditions, operational conditions, structural boundary conditions and instant events, *etc.* For example, the environmental conditions include temperature, humidity and noises, *etc.*, while operational condition includes random dynamic loads, operation speeds, acceleration, vibrations, *etc.* Structural boundary conditions often refer to structural surroundings or connection conditions such as variations of bolts’ tightness degree, thermal expansion and changes of connections between structures, *etc*. No matter what kind of SHM methods are used, almost all the damage monitoring features can be directly affected by the time-varying conditions, which leads to low damage monitoring reliability. Thus a new SHM method should be developed to enhance the damage monitoring reliability of real aircraft structures under time-varying conditions.

Among the existing SHM methods, the Guided Wave (GW) and piezoelectric sensor based SHM method is a promising method because it is a regional monitoring method and is sensitive to small damage. It can be also used on-line both for damage monitoring and impact monitoring [10,11,12,13,14,15,16]. Though many advanced GW and piezoelectric sensor-based damage monitoring methods have been proposed, they mainly concentrate on increasing sensitivity, localization accuracy and damage resolution [17,18,19,20,21,22,23,24,25,26,27]. The basic idea of these methods is still to quantify the GW feature changes caused by damage such as damage scatters, time delays, attenuation, mode conversion and nonlinear properties, *etc.* They can be implemented well in the laboratory under controlled and steady experimental conditions. The environmental condition and the structural boundary conditions are assumed to be constant and the GW signals are acquired under the same operational conditions. However, GW features are vulnerable to changes due to time-varying conditions under structural real service conditions. Particularly, the feature changes introduced by the time-varying conditions are the same as or even larger than the damage-induced changes [7,8]. Several methods including the environmental parameter compensation method [28,29,30,31], baseline signal dependency reduction method [32,33,34,35] and data normalization method [7,36,37,38] have been proposed to deal with the time-varying problem but limitations remain. They still cannot fully realize reliable damage monitoring of real aircraft structures under time-varying conditions at the current stage.

Gaussian Mixture Model (GMM) is a kind of probability and statistics tool for characterizing uncertainties [39,40,41,42]. It can be applied to the problem of modeling arbitrary distributions by decomposing a non-Gaussian distribution into a combination of finite number of Gaussian components (Gaussian distribution) based on unsupervised learning without any *a priori* knowledge. Therefore, some initial studies began to introduce the GMM to the field of SHM to model the uncertainty and non-linearity of sensor signals under time-varying conditions. Tschöpe *et al.* [43] reported the validation of using GMM to classify the degree of damage of a plate-like structure. Banerjee *et al.* [44] used GMM to estimate different crack lengths of a plate-like repaired composite specimen. The time-varying condition of sudden temperature changes was considered in the validation of this method but it is a simple situation. Qiu *et al.* [45] proposed an on-line updating GMM method to monitor a progressive damage on-line. Though the damage monitoring function of GMM was realized and evaluated in the abovementioned studies, they only used basic GMM which was constructed by using the traditional Expectation-Maximization (E-M) method [46]. There are problems that the GMM probability distribution parameters are sensitive to the initialization methods and the number of Gaussian components cannot be changed adaptively during the on-line damage monitoring process. Considering the problems mentioned above, Chakraborty *et al.* [47] proposed a GMM which was constructed by using a Dirichlet process, in which the number of Gaussian components was determined automatically by selecting Gaussian components of high weight. The method was validated on an aluminum structure under cyclic loading conditions. However, a mechanical model of crack propagation needed to be combined with the GMM to realize crack propagation monitoring. This makes the method complicated and an accurate theoretial model of a real complex aircraft structure is difficult to obtain.

All the research mentioned above has shown the capability of using GMM to solve the time-varying problem. However, in depth studies are still needed to enhance the GMM performance for damage monitoring and more validations on aircraft complex structures are also needed.

To enhance the performance of the GMM-based damage monitoring method to deal with the time-varying problem, this paper proposes an effective improved GMM-based damage propagation monitoring method. It can be used on-line without any structural mechanical model. With this method, an on-line adaptive migration mechanism of the GMM is proposed including dynamic learning and Gaussian components split-merge. It is used to migrate the GMM with newly acquired GW monitoring signals during an on-line damage monitoring process to track damage propagation. By using the adaptive migration mechanism, the mixture probability of the GMM and the number of Gaussian components can be optimized on-line adaptively under the situation that the initial GMM is obtained by using the traditional E-M method and the initial number of Gaussian components are fixed. A best match-based Kullback-Leibler (KL) divergence is studied to measure the migration degree of the GMM so as to reveal the weak cumulative variation trend of damage propagation mixed in the time-varying influence. Finally, the method performance is validated on a wing spar of an aircraft.

In the following content, Section 2 proposes the improved GMM based damage propagation monitoring method including the method principle, the basic theory of the GMM, the on-line adaptive migration mechanism of the GMM and the migration degree measuring method. In Section 3, the method is validated on an aircraft wing spar to evaluate the crack propagation monitoring capability under changing structural boundary conditions. Finally, our conclusions are presented in Section 4.

## 2. The Improved GMM-Based Damage Propagation Monitoring Method

### 2.1. Method Principle and Implementation Architecture

**Figure 1:**
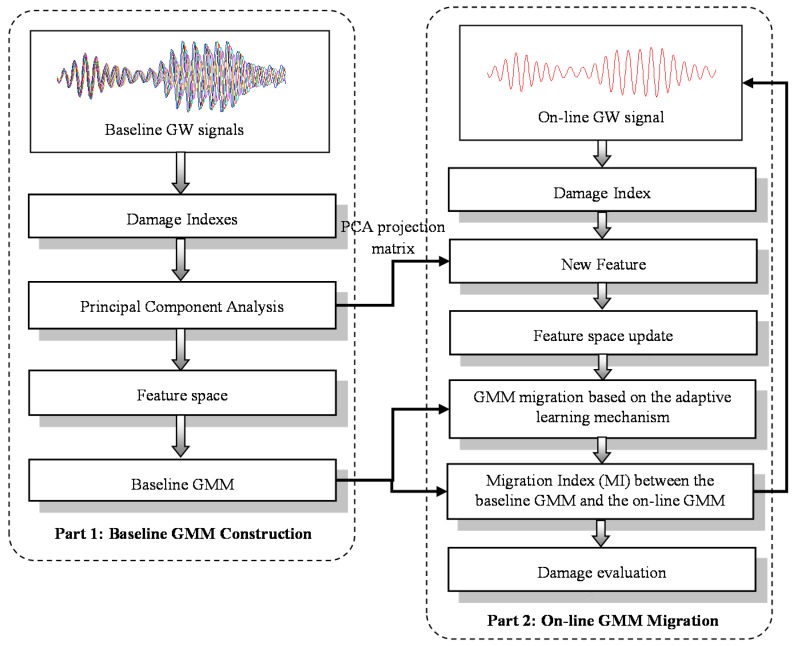
The implementation architecture of the proposed method.

The architecture of the improved GMM based damage propagation monitoring method is given in Figure 1. The method contains two parts:

Part 1 is the baseline GMM construction. In this part, GW signals acquisition is performed under time-varying conditions when the structure is in a healthy state. Damage indexes are calculated based on these GW signals. Then the features, which span the baseline feature space when the structure is healthy, are obtained by fusing the damage indexes based on Principal Component Analysis (PCA) [48]. The principal projection matrix generated by PCA is used to calculate the GW feature in Part 2. At the end, the baseline GMM including the optimized Probability Distribution Parameters (PDPs) is obtained by using the traditional E-M method.

Part 2 is the on-line GMM migration. In this part, once a GW signal is acquired during the on-line damage monitoring process, GW damage indexes can be obtained and the corresponding GW features are obtained by using the principal projection matrix. Then the feature space is updated according to the newly obtained GW features. Though the baseline feature space is covered by the baseline GMM in Part 1, when a new GW feature is obtained in the Part 2, the Gaussian components in the on-line GMM need to be changed to cover the new feature. However the new GW feature is of low proportion compared to the whole feature space. This leads to the fact that the GMM tends to overlap some old regions in the feature space based on those old features. Thus it prevents the on-line GMM from migrating to the new regions to cover the new feature. Thus the feature space is updated first. Then an adaptive migration mechanism including dynamic learning and Gaussian components split-merge is adopted to optimize the probability structure to fulfill fast migration of the GMM on-line. When the PDPs of the on-line GMM are obtained, a Migration Index (MI) is used to measure the distance from the on-line GMM to the baseline GMM.

When the structure is in the healthy state, the GW feature obtained in the on-line damage monitoring process should be covered by the baseline feature space. Thus the PDPs of the on-line GMM and the PDPs of the baseline GMM are overlapped and the MI should be kept at low level. If there is a damage and it propagates gradually, the PDPs of the on-line GMM will show a cumulative migration trend during the on-line adaptive migration process and the MI will show a cumulative increase trend correspondingly. Based on the variation trend of the MI, the reliable damage propagation monitoring under time-varying conditions can be achieved.

### 2.2. Baseline GMM Construction

The GW signals can be considered as a mixture of uncertain changes introduced by time-varying conditions. Consequently a GW feature can be considered a random variable. When the monitored structure is in the healthy state, GW signals are acquired under time-varying conditions and the corresponding features are extracted. **FV** = [***FV***_1_, ***FV***_2_,...., ***FV**_K_*] is denoted as the baseline feature space which is constructed by the features. *K* is the numbers of features in **FV**. ***FV**_k_* = [*F*_1_, *F*_2_,...., *F_d_*] is a *d*-dimensional random variable and represents a feature of **FV**, *k* = 1,2…, *K*. Assuming that **FV** follows a finite mixture distribution and the mixture probability distribution **Ф**(***FV***) can be expressed by a weighted sum of finite Gaussian distributions (Gaussian components) **Ф***_i_*(***FV***), a GMM with is expressed by Equation (1):

(1) $$\Phi \left(\mathrm{FV}|\theta \right)=\sum_{i=1}^{C_{\mathrm{GMM}}}w_{i}\Phi_{i}\left(\mathrm{FV}|\theta_{i}\right)$$

where *w_i_* is the mixture weight and **θ***_i_* is the PDPs of the *i*-th Gaussian component. *C*_GMM_ is the number of Gaussian components. The mean and covariance matrix of the Gaussian component are denoted as ***μ**_i_* and **Σ***_i_* respectively.

Ordinarily, the value of **θ***_i_* is calculated by using traditional E-M algorithm which is consisted of Expectation calculation Step (E-Step) and Maximum likelihood estimation Step (M-Step). The initial value of **θ***_i_* and *w_i_* are obtained by using *k*-means clustering algorithm first. Then in E-Step, the maximum likelihood of each Gaussian component is calculated based on **FV**. In M-Step, the maximum likelihood of each Gaussian component is updated. After multiple iterations between E-Step and M-Step, the optimized **θ***_i_* and *w_i_* are obtained, respectively, and the baseline GMM is constructed.

### 2.3. On-Line Adaptive Migration of the GMM

**Figure 2:**
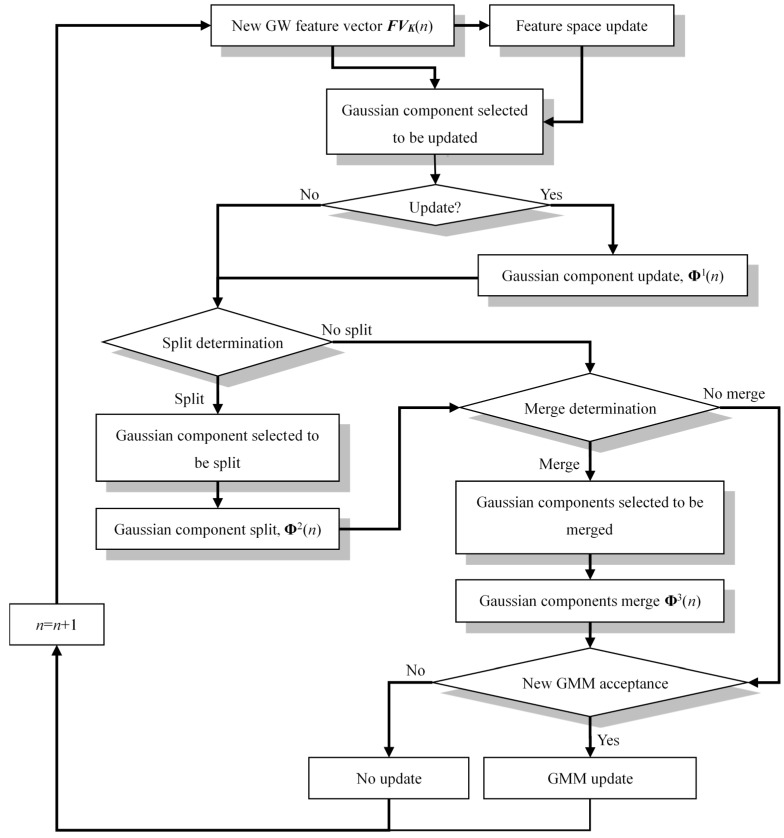
Adaptive migration process of the Gaussian Mixture Model (GMM).

Once a new GW feature is obtained in the on-line monitoring process under time-varying conditions, the feature space **FV**(*n* − 1) is updated to generate a new feature space **FV**(*n*), where *n* (*n* ≥ 1) denotes the on-line monitoring times. Particularly, **FV**(0) represents the baseline feature space. The feature space updating rule is called as moving updating which adds the new feature ***FV**_K_*(*n*) into **FV**(*n* − 1) to be the last (newest) feature and remove the first (oldest) feature of **FV**(*n* − 1). Hence, the number of features in the feature space **FV**(*n*) is still maintained to be *K*. Based on the updated feature space, an on-line adaptive migration mechanism of the GMM is proposed and it is shown in Figure 2.

The migration mechanism is described step by step as follows:

*Step 1: The Gaussian component which should be updated is selected*. The criterion that selects a Gaussian component to be updated is expressed by Equation (2):

(2) $$P_{i}=\left\{ \begin{array}{ll} w_{i}\left(n-1\right)P\left({\mathrm{FV}}_{K}\left(n\right)|\Phi_{i}\left(n-1\right)\right) & \mathrm{if}\;\frac{\left|{\mathrm{FV}}_{K}\left(n\right)-\mu_{i}\left(n-1\right)\right|}{\mathrm{Det}\left(\Sigma_{i}\left(n-1\right)\right)}<\sum_{i=1}^{C_{\mathrm{GMM}}}\frac{1}{C_{\mathrm{GMM}}}\frac{\left|{\mathrm{FV}}_{K}\left(n\right)-\mu_{i}\left(n-1\right)\right|}{\mathrm{Det}\left(\Sigma_{i}\left(n-1\right)\right)}\\ 0 & \mathrm{Others} \end{array}\right.$$

where *P_i_* represents the posterior probability of ***FV**_K_*(*n*) in each Gaussian component, *C*_GMM_ is the number of Gaussian components. **Det** is the determinant value of the covariance matrix. The Gaussian component **Φ***_i_*(*n* − 1) corresponding to the maximum value of *P_i_* is selected to be updated, which means that the distance between ***FV**_K_*(*n*) and **Φ***_i_*(*n* − 1) is the shortest.

*Step 2: Gaussian component update*. The selected Gaussian component is updated based on a recursive filter with two dynamic learning rates which are set to be β(*n*) and ρ [49]. The maximum and minimum value of β(*n*) are β_max_ and *β*_min_. The value of β(*n*) and ρ can be obtained by using Equations (3) and (4):

(3) $$\beta \left(n\right)=\beta_{\max}-\left(\beta_{\max}-\beta_{\min}\right)\left(\exp\left(-\frac{\left\|{\mathrm{FV}}_{K}\left(n\right)-\Phi_{i}\left(n-1\right)\right\|}{\frac{1}{K}\sum_{k=1}^{K}\left\|{\mathrm{FV}}_{k}\left(n-1\right)-\Phi_{match}\left(n-1\right)\right\|}\right)\right)$$

(4) $$\rho =\beta \left(n\right)p\left({\mathrm{FV}}_{K}\left(n\right)|\Phi_{match}\left(n-1\right)\right)$$

where **Φ***_i_*(*n* − 1) is the Gaussian component selected to be updated. **Φ***_match_*(*n* − 1) is the best match Gaussian component corresponding to the feature vector ***FV**_k_*(*n* − 1). ||.|| is Mahalanobis distance. The PDPs of the **Φ***_i_*(*n* − 1) are updated based on Equations (5)–(7). Then a new GMM is generated and it is denoted as **Φ**^1^(*n*).

(5) $$w_{i}\left(n\right)=\left(1-\beta \left(n\right)\right)w_{i}\left(n-1\right)+\beta \left(n\right)$$

(6) $$\mu_{i}\left(n\right)=\left(1-\rho \right)\mu_{i}\left(n-1\right)+\rho \mathrm{FV}\left(n\right)$$

(7) $$\Sigma_{i}\left(n\right)=\left(1-\rho \right)\Sigma_{i}\left(n\right)+\rho \left(\mathrm{FV}\left(n\right)-\mu_{i}\left(n\right)\right){\left(\mathrm{FV}\left(n\right)-\mu_{i}\left(n\right)\right)}^{T}$$

*Step 3: Split determination*. The maximum number and minimum number of Gaussian components are set to be *C*_max_ and *C*_min_, respectively. A uniform random value *r_s_* in the range of [0, 1] is generated. If *r_s_* ≤ 0.5 and *C*_GMM_ < *C*_max_, the GMM **Φ**^1^(*n*) is split based on the following Steps 4 and 5. If *r_s_* > 0.5, the split operation is ignored.

*Step 4: Gaussian component which should be split is selected*. The criterion that selects a Gaussian component to be split is expressed by Equation (8). It represents that the Gaussian component **Φ**^1^*_s_*(*n*) corresponding to the least likelihood value *L*(*s*) needs to be split [50]:

(8) $$L\left(s\right)=\underset{s\in \;C_{AGMM}}{\min}LLP\left({\Phi^{1}}_{s}\left(n\right),\mathrm{FV}\left(n\right)\right)$$

where *LLP* is the likelihood function.

*Step 5: Gaussian component split*. The *w*^2^*_s_*_1_ and **θ**^2^*_s_*_1_ of the **Φ**^2^*_s_*_1_(*n*) and the *w*^2^*_s_*_2_ and **θ**^2^*_s_*_2_ of **Φ**^2^*_s_*_2_(*n*), which are the parameters of the two Gaussian components split from **Φ**^1^*_s_*(*n*) can be calculated based on Equations (9), (10) and (11). Then the updated GMM **Φ**^2^(*n*) is generated:

(9) $${w^{2}}_{s1}\left(n\right)=\beta \left(n\right){w^{1}}_{s}\left(n\right),\;{w^{2}}_{s2}\left(n\right)=\left(1-\beta \left(n\right)\right){w^{1}}_{s}\left(n\right)$$

(10) $${\mu^{2}}_{s1}\left(n\right)=\mathrm{FV}\left(n\right),\;{\mu^{2}}_{s2}\left(n\right)={\mu^{1}}_{s}\left(n\right)+\varepsilon_{1}$$

(11) $${\Sigma^{2}}_{s1}\left(n\right)={\Sigma^{2}}_{s}\left(n\right)+\varepsilon_{2},\;{\Sigma^{2}}_{s2}\left(n\right)={\Sigma^{2}}_{s}\left(n\right)+\varepsilon_{3}$$

where *w*^1^*_s_*, μ^1^*_s_* and **Σ**^1^*_s_* are the parameters of **Φ**^1^*_s_*(*n*). *ε*_1_, *ε*_2_ and *ε*_3_ are small random values in the range of [0, 0.00001].

*Step 6: Merge determination*. A uniform random value *r_m_* in the range of [0, 1] is generated. If *r_m_* ≤ 0.5 and *C*_GMM_ > *C*_min_, the GMM **Φ**^2^(*n*) should be merged based on the following Steps 7 and 8. If *r_m_* > 0.5, the merge operation is ignored.

*Step 7: Gaussian components selected to be merged*. The two Gaussian components **Φ**^2^*_m_*_1_(*n*) and **Φ**^2^*_m_*_2_(*n*) which have the most similar posterior probability are selected to be merged [50]. The selection criterion is expressed by Equation (12):

(12) $$P\left({\Phi^{2}}_{m1}\left(n\right),{\Phi^{2}}_{m2}\left(n\right)\right)=\underset{m1,m2\in \;C_{AGMM}}{\max}P\left(\mathrm{FV}\left(n\right)|{\Phi^{2}}_{m1}\left(n\right)\right)P{\left(\mathrm{FV}\left(n\right)|{\Phi^{2}}_{m2}\left(n\right)\right)}^{T}$$

*Step 8: Gaussian components merge*. The PDPs of the merged Gaussian component **Φ**^3^*_m_*(*n*) can be calculated based on Equations (13)–(15). Then the updated GMM **Φ**^3^(*n*) is generated:

(13) $${w^{3}}_{m}\left(n\right)={w^{2}}_{m1}\left(n\right)+{w^{2}}_{m2}\left(n\right)$$

(14) $${\mu^{3}}_{m}\left(n\right)=\frac{{w^{2}}_{m1}\left(n\right){\mu^{2}}_{m1}\left(n\right)+{w^{2}}_{m2}\left(n\right){\mu^{2}}_{m2}\left(n\right)}{{w^{3}}_{m}\left(n\right)}$$

(15) $${\Sigma^{3}}_{m}\left(n\right)=\frac{\left\{\begin{array}{l} {w^{2}}_{m1}\left(n\right)\left[{\Sigma^{2}}_{m1}\left(n\right)+\left({\mu^{2}}_{m1}\left(n\right)-{\mu^{3}}_{m}\left(n\right)\right){\left({\mu^{2}}_{m1}\left(n\right)-{\mu^{3}}_{m}\left(n\right)\right)}^{T}\right]\\ +{w^{2}}_{m2}\left(n\right)\left[{\Sigma^{2}}_{m2}\left(n\right)+\left({\mu^{2}}_{m2}\left(n\right)-{\mu^{3}}_{m}\left(n\right)\right){\left({\mu^{2}}_{m2}\left(n\right)-{\mu^{3}}_{m}\left(n\right)\right)}^{T}\right] \end{array}\right\}}{{w^{3}}_{m}\left(n\right)}$$

where *w*^2^*_m_*_1_(*n*), μ^2^*_m_*_1_(*n*) and **Σ**^2^*_m_*_1_(*n*) are the parameters of **Φ**^2^*_m_*_1_(*n*). *w*^2^*_m_*_2_(*n*), μ^2^*_m_*_2_(*n*) and **Σ**^2^*_m_*_2_(*n*) are the parameters of **Φ**^2^*_m_*_2_(*n*).

*Step 9: New GMM acceptance*. If the maximum likelihood estimation value of **FV**(*n*) in **Φ**^3^(*n*) is higher than or equal to that of **FV**(*n* − 1) in **Φ**(*n* − 1), the process from Step 3 to Step 8 is accepted as follows:

(1) If split and merge operation are not performed, **Φ**(*n*) = **Φ**^1^(*n*).
(2) If only split operation is performed, **Φ**(*n*) = **Φ**^2^(*n*).
(3) If only merge operation is performed, **Φ**(*n*) = **Φ**^3^(*n*).
(4) If split and merge operation are both performed, **Φ**(*n*) = **Φ**^3^(*n*).

If the maximum likelihood estimation value of **FV**(*n*) in **Φ**^3^(*n*) is lower than that of **FV**(*n* − 1) in **Φ**(*n* − 1) but a uniform random value *r_a_* in the range of [0, 1] is *r_a_* ≤ 0.5, the adaptive learning process is still accepted. If *r_a_* > 0.5, the whole adaptive learning process is ignored and **Φ**(*n*) = **Φ**(*n* − 1) and **FV**(*n*) = **FV**(*n* − 1).

### 2.4. Migration Index

In this paper, a best match Gaussian component-based Kullback–Leibler (KL) divergence [51] is adopted to calculate the MI and it is described as follows. First, the KL divergence between two Gaussian components denoted as **Φ***_i_*(0) and **Φ***_j_*(*n*) is defined based on Equation (16) [52], in which, *μ_i_*(0) and **Σ***_i_*(0) are the parameters of Gaussian component numbered as *i* of the baseline GMM **Φ**(0) and *μ_j_*(*n*) and **Σ***_j_*(*n*) are the parameters of the Gaussian component numbered as *j* of the on-line GMM **Φ**(*n*). **tr** is the matrix trace.

(16) $$\begin{array}{l} D_{KL}\left(\Phi_{i}\left(0\right)\|\Phi_{j}\left(n\right)\right)\\ =\frac{1}{2}\left\{\mathrm{tr}\left[\Sigma_{j}{\left(n\right)}^{-1}\Sigma_{i}\left(0\right)\right]-d-\ln\left[\frac{\mathrm{Det}\left(\Sigma_{j}\left(n\right)\right)}{\mathrm{Det}\left(\Sigma_{i}\left(0\right)\right)}\right]+{\left[\mu_{j}\left(n\right)-\mu_{i}\left(0\right)\right]}^{T}\Sigma_{j}{\left(n\right)}^{-1}\left[\mu_{j}\left(n\right)-\mu_{i}\left(0\right)\right]\right\} \end{array}$$

For each Gaussian component **Φ***_i_*(0) in **Φ**(0), the KL divergence between **Φ***_i_*(0) with all the Gaussian components in **Φ**(*n*) is calculated first based on Equation (16). If the smallest KL divergence happens between **Φ***_i_*(0) and **Φ***_j_*(*n*), it indicates that the best match Gaussian component of **Φ***_i_*(0) in **Φ**(*n*) is **Φ***_j_*(*n*). Based on this point, the best match Gaussian component in **Φ**(*n*) of each Gaussian component of **Φ**(0) can be found. Finally, the best match Gaussian component based KL divergence can be calculated based on Equation (17), in which, *w_i_* and *w_j_* are the mixture weight of the two corresponding Gaussian components of **Φ**(0) and **Φ**(*n*) respectively:

(17) $$\text{MI}\left(\Phi \left(0\right),\Phi \left(n\right)\right)=\sum_{i=1}^{C_{GMM}}w_{i}\;\underset{j}{\min}\left[D_{KL}\left(\Phi_{i}\left(0\right)\|\Phi_{j}\left(n\right)\right)+\ln\frac{w_{i}}{w_{j}}\right]$$

## 3. Method Validation on an Aircraft Wing Spar

### 3.1. Validation Setup

**Figure 3:**
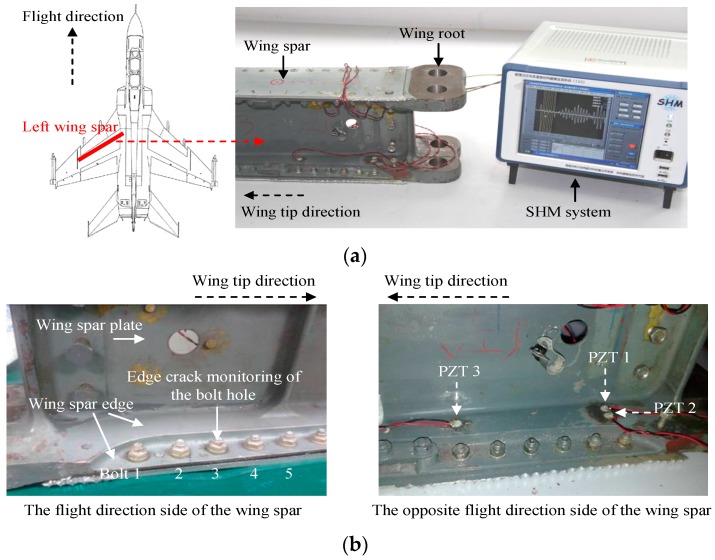

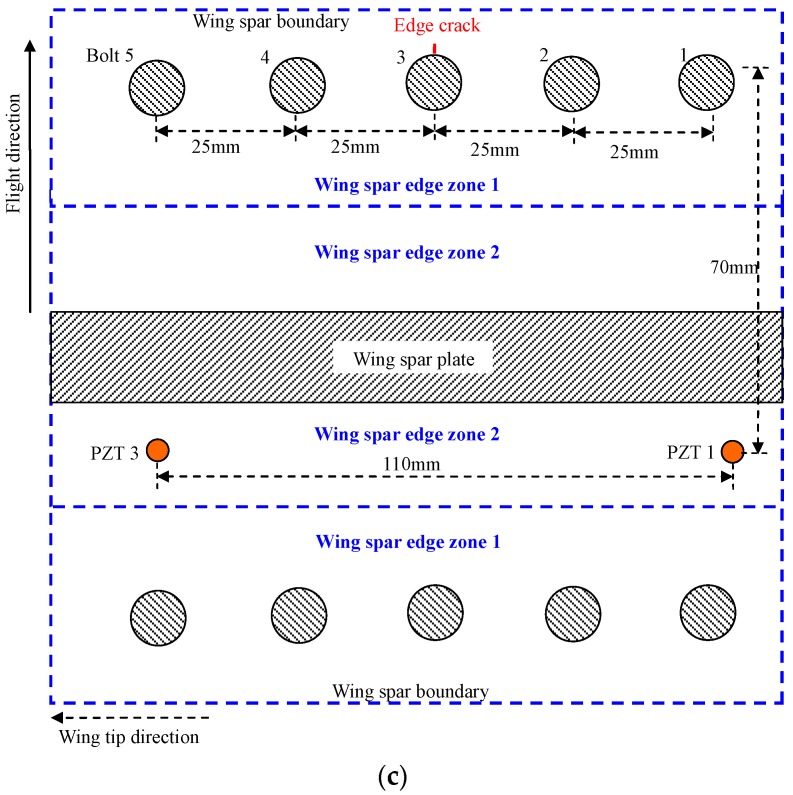
Illustration of the method validation setup. (**a**) The Guided Wave (GW) based Structural Health Monitoring (SHM) system and the aircraft wing spar; (**b**) Wing spar illustration and the piezoelectric sensors placement; (**c**) Schematic diagram of the placement of the piezoelectric sensors.

The validation setup and the GW signal acquisition process are the same as those of a previous study [45]. To keep the integrity of this paper, the validation setup is also described briefly here. As shown in Figure 3, a wing spar which is the primary structure of the aircraft is used to validate the method. It is made of high strength alloy steel and the material is 30CrMnSi. In the spar edge zone 1, the bolts numbered 1–5 are used to validate the method because these bolts suffer from a high fatigue load and they are highly likely to generate bolt hole cracks.

The structure area at the flight direction of the wing spar is very small and sealed. It is an area that Non-Destructive Testing (NDT) cannot cover and no sensors can be placed in it. Thus, only piezoelectric sensors can be placed at the opposite flight direction of the wing spar to monitor any cracks by taking the advantage of the long monitoring range of the GW and piezoelectric sensor-based SHM method. The piezoelectric sensors numbered PZT 1 and PZT 3 on the spar edge zone 2 are used in the validation to excite and receive GW signals. The thickness of spar edge zone 1 is 20.8 mm. The thickness of spar edge zone 2 is 9.70 mm. Thus the thickness of the structure is suddenly changed between the two zones. There is a spar plate of thickness 16 mm placed between the piezoelectric sensors and the five bolts. The thickness is also suddenly changed in this area. Thus the wing spar is a typical complex structure for the GW and piezoelectric sensor based SHM method. An edge crack is produced at bolt hole 3 to simulate the fatigue damage. It can introduce GW reflections in the bolt hole crack, the spar plate, the structural boundary, all the bolts and the areas whose thickness changes suddenly. The reflection introduced by the crack is called damage reflection and the reflections introduced by all the structural factors are considered to be boundary reflections. Thus this validation is used to test the crack propagation monitoring capability of the improved GMM-based method under changing structural boundary conditions. It is simulated by loosening and fastening the bolts to different tightness degrees.

A GW and piezoelectric sensor-based SHM system shown in Figure 3a [53] is used to excite and receive GW signals. The excitation signal is a modulated five-peak sine signal with the central frequency of 200 kHz [23]. The sampling rate and excitation amplitude are set to be 10 MSamples/s and ±70 V respectively. To validate the method which includes two parts, the GW signal acquisition process is performed to be the following two parts correspondingly. In Part 1, GW signals are acquired based on the following four steps:

*Step 1*: Acquire a GW baseline signal when all the bolts are tight.
*Step 2*: Loosen one bolt and acquire a GW signal, and then fasten the bolt and acquire a GW signal.
*Step 3*: Repeat this process on each bolt respectively.
*Step 4*: Repeat Steps 2 and 3 twice.

Therefore, one GW baseline signal and the other 30 GW signals are obtained in Part 1. They are numbered as S_1_–S_31_ according to the acquisition sequence. In Part 2, the GW signals are acquired basd on the following steps:

*Step 1*: Repeat the Steps 2 and 3 in Part 1 twice.
*Step 2*: Remove the bolt 3 and produce a crack of length 1 mm at the bolt hole. Then fasten the bolt 3 and repeat Step 1.
*Step 3*: Remove the bolt 3 and extend the crack length to 2 mm. Then fasten the bolt 3 and repeat Step 1.
*Step 4*: Remove the bolt 3 and extend the crack length to 3 mm. Then fasten the bolt 3 and repeat Step 1.

Therefore, 80 GW signals are obtained. According to the acquisition sequence, the 80 GW signals are numbered as S_32_–S_111_.

### 3.2. GW Signals and Damage Index

**Figure 4:**
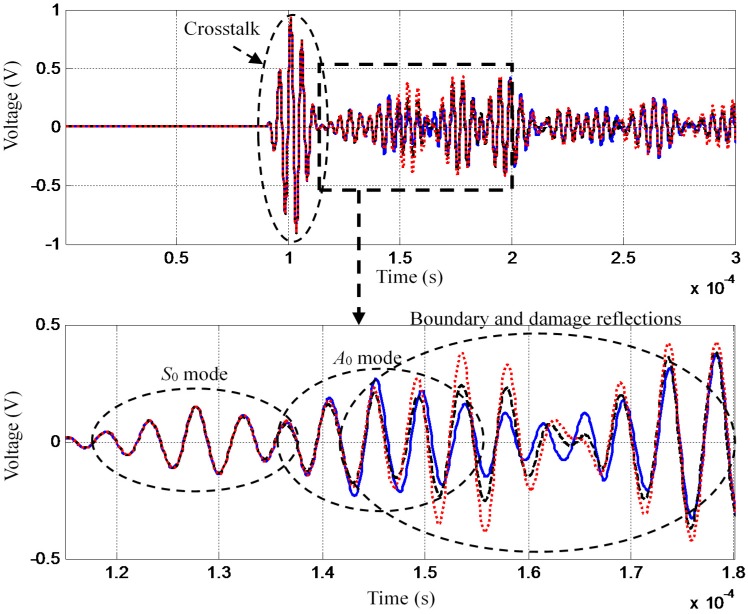
Typical GW signals acquired under changing structural boundary conditions.

The boundary condition variation can influence the GW boundary reflections. In the validation, the bolts’ degree of tightness changes are regarded to be the changing structural boundary conditions as mentioned before. Figure 4 shows three typical GW signals acquired under three situations including structural healthy condition without bolt loosing (blue line), structural healthy condition with bolt loosing (red dashed line) and the crack of length 1 mm with bolt loosing (black dotted line) respectively. 

It indicates that the time domain variation of the GW signals introduced by the changing structural boundary condition is larger than that caused by the crack propagation. Detailed information on the time-varying influence of the GW signals can be found in the previous study [45]. In this paper, the GW signal in the time segment from 1.15 × 10^−4^ s to 1.8 × 10^−4^ s is used. The crosstalk shown in the GW signals is introduced by the SHM system.

**Figure 5:**
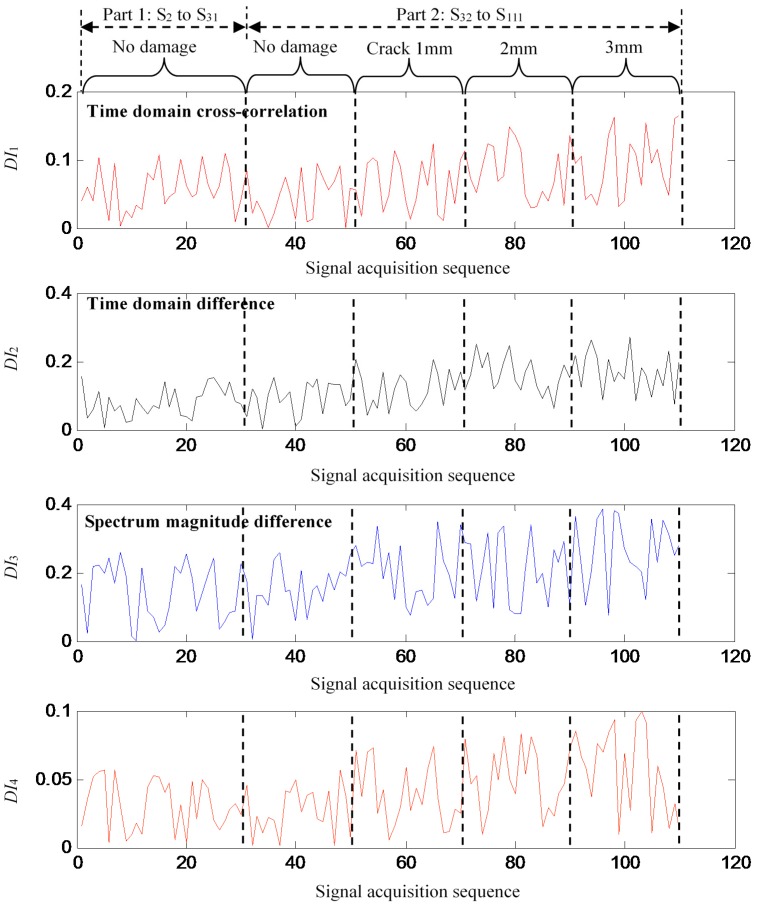
Damage indexes extracted from GW signals S_2_–S_111_.

The damage indexes of GW signals S_2_–S_111_ are calculated based on the baseline signal S_1_ and they are shown in Figure 5. It can be noted that the crack propagation is difficult to evaluate based on these damage indexes because of the large random variations introduced by the changing structural boundary conditions.

### 3.3. GMM On-Line Adaptive Migration

**Figure 6:**
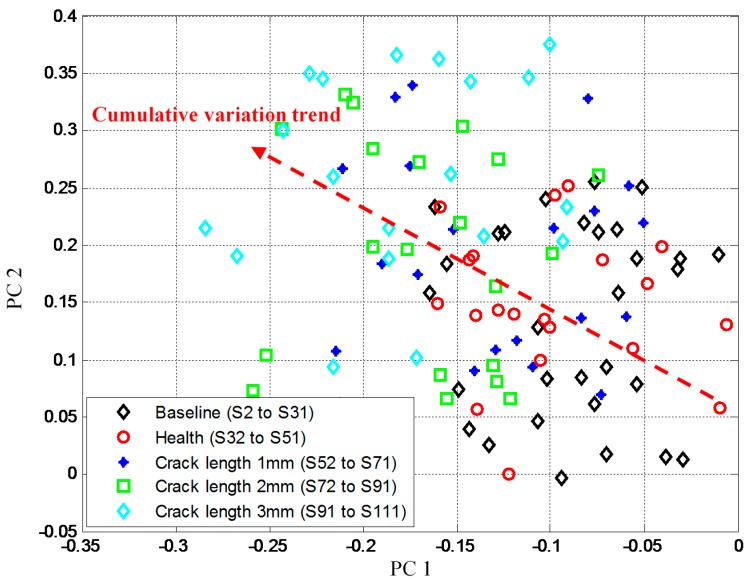
GW features obtained by using PCA.

The damage indexes of S_2_–S_31_ are used to construct the two-dimensional baseline feature space by using PCA and the corresponding PCA projection matrix is obtained. The product of S_32_–S_111_ and the PCA projection matrix is performed to get the corresponding GW features. All these GW features are shown in Figure 6. Though the changing structural boundary condition introduce large random variation to the damage indexes, The GW features still show a weak cumulative variation trend caused by the crack propagation.

**Figure 7:**
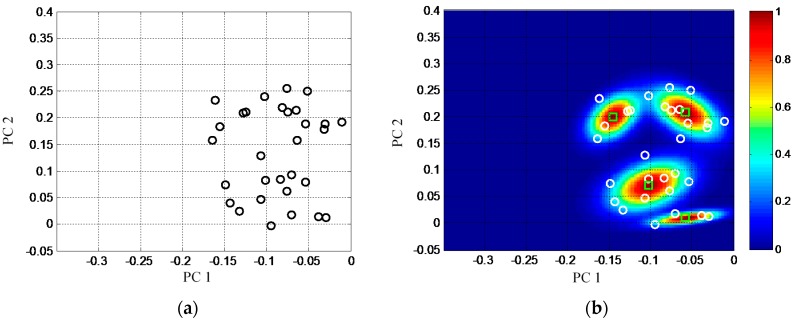
Demonstration of the baseline features and the baseline GMM. (**a**) The baseline features; (**b**) The baseline GMM.

In the method of Part 1, the baseline GMM is shown in Figure 7b. It is constructed based on the baseline features shown in Figure 7a. The number of Gaussian components is set to be *C*_GMM_ = 4 initially. The GMM is expressed by a nebula image. Each nebula is a Gaussian component and the color is the normalized mixture probability density of the Gaussian component. It can be seen that the baseline GMM covers all the baseline features well.

**Figure 8:**
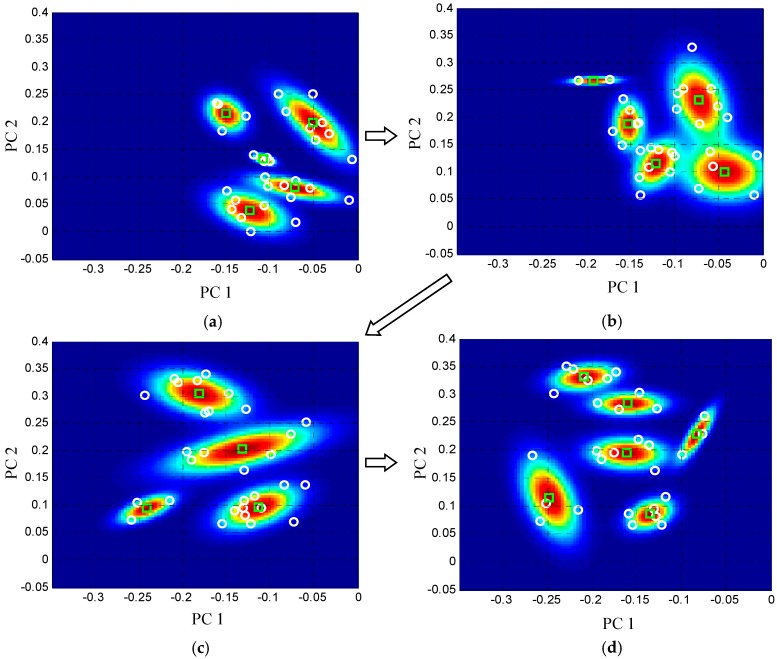
Demonstration of the adaptive migration process of the GMM. (**a**) On-line GMM of healthy state; (**b**) On-line GMM wiht crack of length 1 mm; (**c**) On-line GMM with crack of length 2 mm; (**d**) On-line GMM with crack of length 3 mm.

In the method of Part 2, the on-line GMM should monitor the crack propagation reliably based on the 80 GW features of S_32_–S_111_. Thus the 80 GW features are input to the Part 2 one by one according to the acquisition sequence to update the on-line GMM based on the adaptive migration mechanism. The limitations of the learning rate are set to be *β*_max_ = 0.1 and *β*_min_ = 0.01, respectively. The limitations of the number of Gaussian components are set to be *C*_max_ = 6 and *C*_min_ = 2, respectively. The number of features in the feature space is set to be *K* = 30. Figure 8 shows some typical on-line GMMs under the four situations including structural health condition, crack propagation length 1 mm, 2 mm and 3 mm, respectively. It shows that the number of Gaussian components is changed to cover the GW features of different feature spaces and the on-line GMM shows a migration trend accompanying the crack propagation. The migration results indicate that the on-line GMM can track the weak cumulative variation trend of feature space by changing the structure of Gaussian mixture probability adaptively and this will lead to a cumulative increase of the MI.

### 3.4. Crack Propagation Monitoring Results

**Figure 9:**
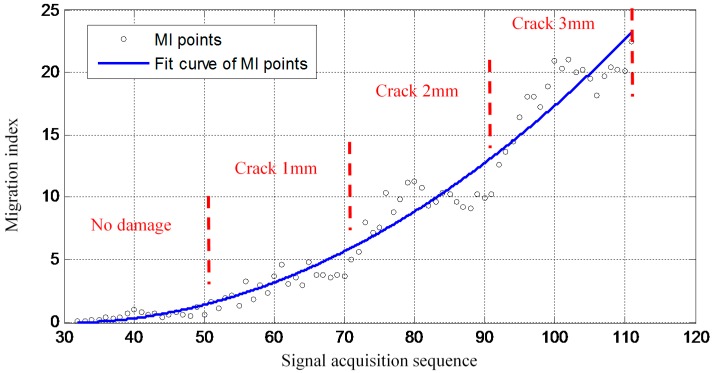
The crack propagation monitoring result of the proposed method.

The MI result obtained based on the 80 GW features of S_32_–S_111_ is shown in Figure 9. There is no damage at the beginning stage of the on-line migration of the GMM. Thus the MI remains at a low level. This indicates that the random structural boundary variations have little influence on the MI when the wing spar is in the healthy state. When there is a crack at the bolt hole and it propagates, the MI increases gradually accompanying the crack propagation though the influences of the changing structural boundary condition of the GW signals are large. The increasing trend is slowed down at each crack length when the crack is not propagated. This is because that the damage-induced effect becomes weak gradually accompanying the accumulation of the features with the same damage influence. When the crack length is propagated, the MI increases again. Therefore, the increasing of the MI shows three steps corresponding to the three different crack propagation length. 

**Figure 10:**
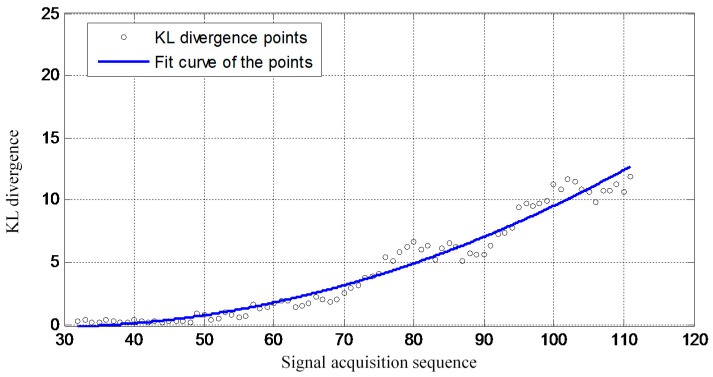
The crack propagation monitoring result by using the previous method [45].

**Figure 11:**
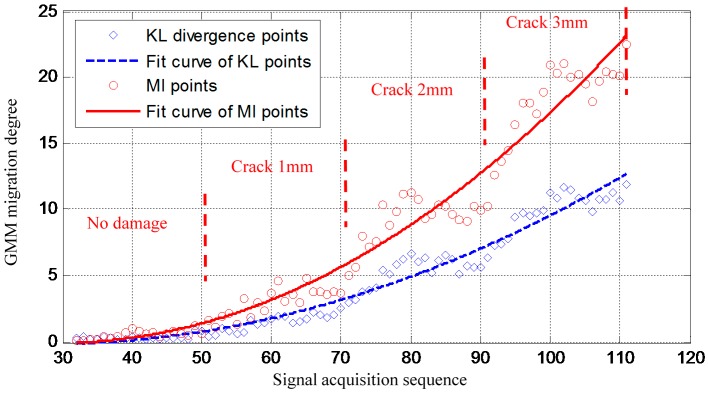
The comparsion of the crack propagation monitoring results between the two methods.

Overall, the validation results show that the proposed improved GMM method is capable of monitoring damage propagation under time-varying conditions. Figure 10 gives a crack propagation monitoring result obtained by using the previous method [45]. In that method, the migration of the GMM is quantified by using the ordinary KL divergence. Thus the longitudinal coordinate of Figure 9 and Figure 10 are labeled as Migration index and KL divergence, respectively. To have a better comparison, the crack propagation monitoring results of the proposed method and that of the previous method are drawing in the same figure as shown in Figure 11.

It can be noted from Figure 11 that the MI increases more dominantly and faster than the ordinary KL divergence. This indicates that the newly proposed method has higher damage sensitivity than the previous method. This is because that the KL-divergence is calculated by using the PDFs of the two GMMs directly in the previous study, in which, the mean and the covariance matrix of the GMM are obtained by using the weighted sum of the mean and the weighted sum of the covariance matrix of Gaussian components. It is a kind of averaging effect which leads to a reduction effect of GMM migration measuring. In addition, if the two curve fits of the points in Figure 11 are considered to be two signals, respectively, and the deviation of the points from two curve fits are considered to be the noise, then the signal-to-noise ratio of the MI is lower than that of the KL-divergence under the situation that the GMM with large migration. It means that the KL-divergence is more stable than the MI under that situation because of the averaging effect mentioned above.

## 4. Conclusions

This paper proproses an improved GMM-based damage propagation monitoring method. With this method, the baseline GMM is constructed only based on the traditional E-M method which includes a large amount of iteration steps in Part 1, but the updating of the on-line GMM only depends on the adaptive migration mechanism. Thus, the whole process of the method is highly efficient. Though the number of Gaussian components is intially fixed in the E-M algorithm in Part 1, the on-line GMM can be updated adaptively including the number of Gaussian components and PDPs. It only depends on the nature of the feature space without any *a priori* knowledge of damage and time-varying conditions. In addition, the improved GMM is a kind of data-driven based probability and statistical model. No mechanical models of the damage and structure are needed. 

An aircraft wing spar is used to validate the method. The validation results show that the crack propagation monitoring under changing structural boundary conditions can be monitored reliably by using the proposed method based on the cumulative increasing trend of the MI. 

More validations on real aircraft structures will be performed in the near future to validate the method under more complicated time-varying conditions. In order to evaluate the damage occurrence and propagation length, the normalization and quantification of the MI should be also further studied.

Although this method has only been validated on an aircraft structure of alloy steel, it is not limited by the properties of the structure. In recent decades, composite materials have been gradually used on important aircraft load-bearing structures. The damage monitoring of aircraft composite structures has thus become an important topic in the SHM research field. Thus method validation on real aircraft composite structures will be also performed in the near future.
